# A Word with Our Readers

**Published:** 1891-09

**Authors:** 


### HALL'S
### JOURNAL OF HEALTH

TRUTH DEMANDS NO SACRIFICE; ERROR CAN MAKE NONE.

Vol. 38. SEPTEMBER, 1891. No. 9.

### A WORD WITH OUR READERS.

Some of our readers are under the impression that this Journal is conducted by some present Dr. Hall, who is a medical specialist or holds a proprietary interest in some one or more of its advertised remedies. Only a day or two ago, we received a communication from a fair subscriber, addressed to Doct. Hall, of HALL'S JOURNAL OF HEALTH, and saying among a variety of pleasant things, "I am now following your system of treatment."

Now this is all a mistake. Neither the Editor in Chief or any of the staff of the journal, use its columns to urge upon its readers any system of treatment or cure from which they derive any pecuniary advantage.

For nearly forty years the JOURNAL has been a monthly visitor in thousands of families. Its aim has always been to point out the surest means of attaining and preserving good health, the elements of which are simple and natural.

Plain nutritious food, taken in moderation at regular periods, pure air and water, with open air exercise, will obviate the necessity of continually bolstering and patching a pampered, over-fed and over-indulged physique; but sometimes in spite of every precaution, disorders will gain the upper hand and it becomes necessary to assist nature in her endeavor to preserve or restore a just equilibrium of the various organs upon whose regular and vigorous action good health depends.

It is then that the advice of a competent physician should be secured and followed till the cause of the disease is eradicated, and impaired health restored.

There are, however, many ailments, common to both old and young, which only require good nursing and such correctives as almost every family have in store, "old women's remedies," as they are termed, but the "old women" are often wiser than the doctors, and their remedies never kill, if they fail to cure.

Having set the JOURNAL right with its readers on the drug question, we would call attention to our new Premium List, open equally to new subscribers and renewals, which to say the least, is something extraordinary.

